# Short- and Long-Term Consequences of Late-Preterm and Early-Term Birth

**DOI:** 10.3390/children12070907

**Published:** 2025-07-09

**Authors:** Muhammad Arham, Katarzyna Wróblewska-Seniuk

**Affiliations:** 1 Department of Pediatrics, Rawalpindi Medical University, Rawalpindi 46000, Pakistan; arham362@gmail.com; 2 II Department of Neonatology, Poznan University of Medical Sciences, 60-535 Poznan, Poland

**Keywords:** late preterm, early term, outcomes, morbidity, mortality, risk factors, epidemiology

## Abstract

Late-preterm (34^0/7^–36^6/7^ weeks) and early-term (37^0/7^–38^6/7^ weeks) newborns were, up until recently, erroneously categorized as low-risk and were conflated with full-term (39^0/7^–40^6/7^ weeks) deliveries. However, emerging evidence refuted this notion and demonstrated that late-preterm and, to a lesser extent, early-term newborns have a significantly higher risk of experiencing various neonatal morbidities, including respiratory distress syndrome, transient tachypnea of the newborn, pneumonia, jaundice, hypoglycemia, and breastfeeding difficulties, compared to their full-term counterparts—reflecting their relative physiologic and developmental immaturity. Recent evidence also unravels the lingering adverse effects of late-preterm and early-term delivery up until mid-adulthood, with the increased susceptibility of these newborns to neurodevelopmental delays, behavioral and neuropsychiatric problems, and adult chronic diseases. Moreover, apart from increased neonatal and infant mortality rates, these newborns continue to encounter a heightened risk of mortality even up to mid-adulthood. As the full spectrum of the complications these newborns face is gradually being unveiled, this review presents and discusses the current knowledge base, identifies gaps in the literature, and highlights future research implications.

## 1. Introduction

With an estimated annual rate of 13.4 million newborns delivered before 37 completed weeks of gestation, preterm birth represents a grave global health challenge and is the leading cause of neonatal and child mortality [1,2,3]. Since the morbidity and mortality rates of preterm delivery increase exponentially as gestational age decreases, medical professionals have for decades prioritized infants born <32 weeks of gestation while erroneously categorizing all deliveries occurring beyond this hallmark as low-risk [4,5]. Specifically, newborns delivered after 34 weeks were considered relatively mature and healthy and treated along the same lines as term infants. It was not until recently that mounting evidence challenged this paradigm and identified late-preterm (34^0/7^–36^6/7^ weeks) and, subsequently, early-term (37^0/7^–38^6/7^ weeks) births to be not completely out of the danger zone [6].

Although late-preterm newborns appear larger than those born before 34 weeks, it is now known that they are physiologically and developmentally not fully mature [6,7]. This understanding has been substantiated by the accumulating literature, which has highlighted that late-preterm birth is associated with an increased risk of various neonatal complications, including respiratory morbidities, hypoglycemia, hypothermia, hyperbilirubinemia, neurologic problems, and infections, in addition to higher rates of readmission and mortality compared to term deliveries [8,9,10,11]. These alarming revelations paved the way for these newborns to be identified as a new high-risk subgroup in neonatology.

More recently, researchers have delineated the heterogeneity of outcomes even within the ‘term’ window and furnished clear evidence of poorer neonatal and infant outcomes for early-term newborns compared to those delivered at 39^0/7^–40^6/7^ weeks of gestation, known as full-term infants [6,12,13,14]. Although these adverse outcomes are not as severe as those experienced by late-preterm newborns, they nevertheless include respiratory problems, jaundice, NICU admission, and a modest increase in neonatal and infant mortality risk, underscoring the persistent threat posed by any delivery occurring before full term [13,14].

Apart from the increased risk of neonatal complications, emerging evidence has begun to unravel the lingering adverse effects of late-preterm and early-term birth into early childhood, through adolescence, all the way up to mid-adulthood. In addition to various medical and neurodevelopmental problems encountered during the later years of life, late-preterm and early-term newborns experience a heightened risk of mortality even as adults when compared to full-term births [15,16,17,18].

With advances in pediatric research, our knowledge of late-preterm and early-term newborns continues to evolve, and the full extent of the effects of being born in that gestational age window is gradually being uncovered. The latest research has not only provided better insights into the broad range of short- and long-term complications faced by these newborns but also discerned various risk factors of morbidity, warranting an up-to-date review of the literature to reflect the current challenges faced by infants born before full term.

In this narrative review, we strive to present a comprehensive analysis of the short- and long-term morbidities associated with late-preterm and early-term birth, with the rationale to streamline healthcare provision for this vulnerable population and equip medical professionals with the knowledge needed to anticipate risks and implement timely intervention. This review will discuss the definition, prevalence, risk factors, and the short- and long-term consequences of late-preterm and early-term birth, along with identifying new research directions.

## 2. Definition

Although preterm delivery has long been classified as birth <37 completed weeks of gestation, the last century saw newborns born at 33 weeks through term being described with a plethora of terminology, including ‘near term’, ‘marginally preterm’, and ‘mildly preterm’, among others. These descriptors carried a false implication that these newborns are almost identical to term births and hence made ground for clinicians to overlook the risks associated with these deliveries.

In 2005, an expert workshop sponsored by the National Institute of Child Health and Development (NICHD) acknowledged the negative impact of these misclassifications, underscoring how these various terminologies failed to reflect the actual risk associated with these births. Therefore, the panel decided to classify newborns born between 34^0/7^ and 36^6/7^ weeks of gestation as ‘late preterm’ to convey their vulnerability appropriately [19]. This classification was readily adopted by the American Academy of Pediatrics (AAP) in 2007 [20].

The descriptor ‘early term’ was coined more recently. Conventionally, term birth was broadly defined as delivery at 37–42 weeks of gestation. The 2012 pregnancy workgroup emphasized that the heterogeneous outcomes of births within this 6-week gestational age window warranted sub-classification of ‘term’ birth. The workgroup recommended designating births at 37^0/7^–38^6/7^ weeks of gestation as early term, 39^0/7^–40^6/7^ weeks as full term, and 41^0/7^–41^6/7^ weeks as late term, hence discouraging ‘term’ as a standalone definition [21]. The American College of Obstetricians and Gynecologists followed suit and endorsed this new subcategorization to elicit refinement of healthcare delivery and research for term newborns [22].

However, it is worth noting that any gestational age classification system utilized is bound to be arbitrary and reliant on the accuracy of gestational age estimation. The narrow differences between various gestational age subgroups might fall within the inherent variability of any gestational age calculation—a dilemma experts should address.

## 3. Prevalence

In 2020, the reported worldwide prevalence of preterm deliveries was 9.9%, with the highest rates recorded in southern Asia (13.2%) [1]. The US preterm birth rate in 2021 was estimated to be 10.49%, the highest since 2007. Nearly seventy-five percent of these preterm births were late preterm, representing 7.67% of total live births. Early-term newborns represented 28.76% of all live births and nearly one-third of all term deliveries. Over the past decade, the birth rates of late-preterm and early-term newborns in the US have increased by around 10% [23]. Moreover, 90% of the total increase in US preterm births between 2014 and 2019 has been attributed to the rise in late-preterm deliveries [24].

Other developed countries in the West have late-preterm and early-term birth rates ranging from 3 to 6% and 15 to 30%, respectively [25,26]. Analogous to the trend in the US, Iceland witnessed an uptick in late-preterm deliveries between 1997 and 2016 [27]. The birth characteristics data of England and Wales recorded 34,183 late-preterm and 153,105 early-term deliveries in 2022, representing nearly 31% of all live births [28]. Similar rates are observed in other parts of the world, like the Middle East, China, and Brazil. A Qatari population-based register study revealed that late-preterm and early-term newborns represented 6.4% and 33.7% of all live births, respectively [29]. Late-preterm births in China (5.17% of total live births) constitute 75% of all preterm deliveries and have increased by 8.8% between 2012 and 2018 [30]. The rate of early-term deliveries is around 24% in China and 35% in Brazil [31,32].

On the other hand, low- and lower-middle-income nations lag behind high-income countries in furnishing sufficient data regarding late-preterm and early-term deliveries. This scarcity of data hinders our ability to comprehend the global burden of these vulnerable newborns. Considering that preterm birth, in general, is much more common in the developing world, it can be extrapolated that the rates of late-preterm deliveries, and to some extent, early-term births, are bound to be much higher in these countries compared to high-income nations [1]. Some limited evidence illustrates the gravity of the prevailing circumstances in resource-poor parts of the world, like southern Asia, where late-preterm births constitute up to 12.7% of all live births [33].

Most prevalence estimates have used singleton deliveries to calculate late-preterm and early-term birth rates, often ignoring multiple births from the equation. However, this factor significantly influences the birth rates of these newborns [34,35]. A preponderance of multiples is born before term, accounting for about twenty percent of all late-preterm births and five percent of all early-term deliveries [34]. China recently witnessed a dramatic increase in preterm births among multiple pregnancies, more due to late-preterm than very-preterm deliveries [30]. Multiple gestation has also been cited as one of the factors propelling the uptick in late-preterm and, by extension, early-term deliveries observed over the past two decades [19,20].

## 4. Risk Factors

Nearly two-thirds of preterm deliveries occur spontaneously, while the remaining one-third are due to obstetric intervention [36]. Although factors associated with preterm birth, in general, have been extensively described in the literature [37], relatively fewer studies have investigated the specific risk factors for late-preterm and early-term delivery; however, the available evidence unveils many shared mechanisms [38,39,40].

Various maternal, fetal, obstetric, sociodemographic, and environmental factors have been implicated in late-preterm and early-term delivery (Table 1). Brown et al. [38] identified diabetes mellitus, hypertension, eclampsia/preeclampsia, placentation disorders, poly-/oligohydramnios, and a previous preterm delivery as critical factors leading to spontaneous late-preterm and early-term delivery. In contrast, infection and inflammation were associated only with late-preterm birth in their cohort. Evidence has also shown that apart from previous preterm delivery, even prior delivery of an early-term infant can increase the risk of subsequent late-preterm and early-term birth [41]. A study conducted on a Middle Eastern population revealed that low maternal education and chromosomal/congenital abnormalities were associated with late-preterm delivery; parity ≥1 and newborn male gender with early-term delivery; and advanced maternal age and assisted pregnancy with both late-preterm and early-term delivery [29].

Assisted reproductive technology has also surfaced as an independent risk factor for late-preterm and early-term birth [42]. Other contributing factors include low maternal BMI [43], an inter-pregnancy interval of <12 months [44], antenatal exposure to air pollutants [45,46] (the third trimester being the most susceptible window [47]), exposure to high ambient temperatures during pregnancy [48], maternal drug abuse [49], and trait anxiety [50].

The increasing trend in provider-initiated deliveries is believed to be driving the corresponding increase in late-preterm birth rates [24]. Cesarean section increases the risk of late-preterm [51] and early-term birth [52], and interestingly, a previous cesarean delivery is associated with an increased odds of a subsequent pregnancy being late preterm [53]. Moreover, recent evidence unearthed an association between term cesarean in the second stage of labor and the risk of late-preterm and early-term birth in a subsequent pregnancy, illustrating the complexity of the underlying mechanisms [54].

Certain maternal medical conditions have been associated with a greater risk of medically indicated late-preterm and early-term delivery, including diabetes mellitus, hypertension, preeclampsia/eclampsia, and placental abruption [39]. On the other hand, respiratory, gastrointestinal, and hormonal disorders have been exclusively implicated in medically indicated early-term delivery [39].

## 5. Neonatal Morbidity

**Figure 1:**
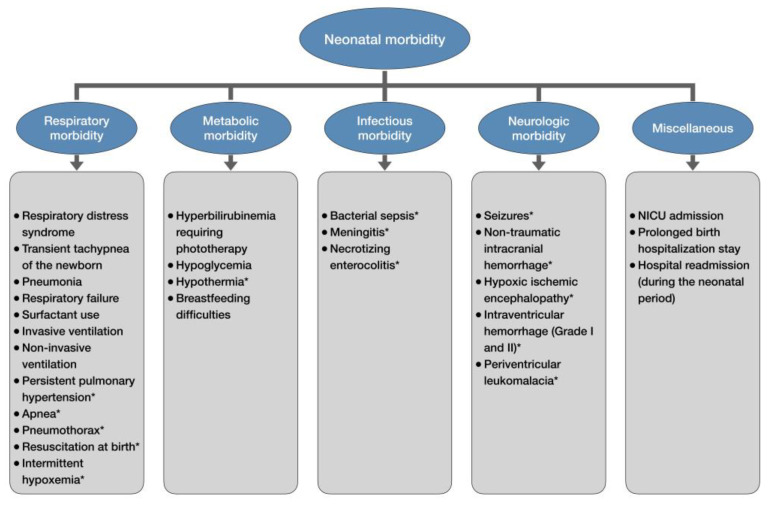
Neonatal morbidity of late-preterm and early-term newborns. * only late-preterm newborns.

Late-preterm and early-term infants experience higher rates of morbidities during birth hospitalization than full-term newborns, including respiratory, metabolic, infectious, neurologic, and various other pathologies (Figure 1) [14,55,56]. As the gestational age decreases below 39 weeks, neonatal morbidity and hospitalization rates increase exponentially [57,58]. A large population-based study reported the neonatal morbidity risk at 39, 38, 37, 36, 35, and 34 weeks of gestation to be 2.6%, 3.3%, 5.9%, 12.1%, 25.6%, and 51.7%, respectively [57]. Similarly, the birth hospitalization rates increase sharply from 5.8% at 39–41 weeks to 96.9% at 34 weeks [58]. The duration of initial birth hospitalization also tends to be longer in late-preterm newborns compared to full-term births [55]. The mean length of hospital stay for neonates born at 34, 35, and 36 weeks of gestation is 12.6, 6.1, and 3.8 days, respectively [59].

### 5.1. NICU Admission

The predisposition of late-preterm and early-term neonates to encounter severe morbidities translates into these newborns requiring more frequent and more prolonged admission to the neonatal intensive care unit (NICU) [60]. A Canadian retrospective cohort study concluded that, compared to infants delivered full term, late-preterm newborns were six times more likely to require NICU triage/admission, whereas early-term infants had a 54% increased risk [56]. This study also identified placental ischemia and other hypoxia as exacerbating factors for this risk association. Fayed et al. [61] reported that both late-preterm and early-term newborns have an almost similar increased risk of NICU admission compared to their full-term counterparts (67% and 57%, respectively).

The mode of childbirth also seems to influence the risk of requiring neonatal intensive care. In a multicenter cohort, spontaneous late-preterm newborns had around four times the risk of requiring intensive care compared to controls delivered at full term. On the other hand, provider-initiated late-preterm births had around fifteen times the risk, suggesting a potential additive effect of various risk factors [62]. Similarly, resuscitation at birth has also been shown to mediate the impact of gestational age on NICU admission risk. Spillane et al. [63] assessed the outcomes of those late-preterm and early-term newborns who were admitted to the well-baby nursery after resuscitation at birth. The risk of subsequent transfer to the NICU was significantly higher in both groups than in neonates delivered at 39 weeks of gestation (RR for 35, 36, 37, and 38 weeks: 8.94, 8.26, 4.63, and 4.51, respectively).

### 5.2. Respiratory Morbidity

Late-preterm and early-term newborns more frequently encounter various respiratory complications, including respiratory distress syndrome, transient tachypnea of the newborn, pneumonia, and respiratory failure, compared to full-term births [60,64]. Brown et al. [56] reported that the risk of neonatal respiratory morbidity was higher in late-preterm (RR = 6.16, 95% CI: 5.39–7.03) and early-term (RR = 1.46, 95% CI: 1.29–1.65) newborns compared to full-term neonates. A study of an Italian cohort of 14,515 infants born between 34 and 41 weeks of gestation demonstrated that the adjusted odds of composite respiratory morbidity increased from 3.3 (95% CI: 2.0–5.5) at 37 weeks to 40.8 (95% CI: 19.7–84.9) at 34 weeks of gestation, compared to newborns between 39 and 41 weeks [64].

The Consortium on Safe Labor study analyzed the respiratory morbidity of 233,844 infants delivered between 34^0/7^ and 40^6/7^ weeks of gestation across nineteen US hospitals [60]. Respiratory distress syndrome was the most common respiratory morbidity experienced by late-preterm and early-term newborns, with an incidence rate as high as 10.5% at 34 weeks, decreasing to 0.3% at 38 weeks of gestation. From 34 to 38 weeks, the odds of respiratory distress syndrome decreased with each advancing week compared with 39 to 40 weeks [34 weeks: aOR 40.1 (95% CI 32.0–50.3); 35 weeks: 21.9 (17.8–26.9); 36 weeks: 9.1 (7.5–11.1); 37 weeks: 3.1 (2.5–3.7); 38 weeks: 1.1 (0.9–1.4)]. A similar trend was also observed for transient tachypnea of the newborn, pneumonia, respiratory failure, and surfactant and ventilator use, demonstrating a dose–response relationship between gestational age and respiratory morbidity. Interestingly, the odds of all respiratory complications at 38 weeks were not statistically different from full-term controls, questioning the construct of grouping all early-term newborns in the same risk category.

The frequency of severe respiratory disorders requiring non-invasive or invasive ventilation is much higher in late-preterm newborns (8.31%) and, to a lesser extent, in early-term newborns (0.84%) compared to full-term neonates (0.28%) [58]. Late-preterm neonates are notably more likely to be placed on high-frequency oscillatory ventilation compared to newborns between 37 and 41 weeks of gestation (6.4% vs. 0.7%) and require prolonged intubation (median 5.0 vs. 2.0 days) [65].

The susceptibility of late-preterm infants to various other pulmonary pathologies like persistent pulmonary hypertension (RR 4.9), apnea (RR 15.7), and pneumothorax (RR 3.4) is much higher compared to neonates >37 weeks of gestation [66]. Moreover, late-preterm newborns have a four-fold risk of having an APGAR < 7 at birth compared to full-term neonates [61] and a three-fold risk of being subjected to resuscitation compared to neonates between 37 and 40 weeks [51]. Intermittent hypoxemia also more frequently occurs in late-preterm newborns compared to full-term controls [67].

Lately, investigators have been studying pulmonary complications in late-preterm and early-term newborns delivered via elective cesarean section. Evidence demonstrates that elective cesarean sections performed at ≤38^6/7^ weeks have twice the risk of newborn respiratory morbidity compared with ≥39^0/7^ weeks [68]. A study evaluating a Japanese cohort of elective cesarean deliveries at term calculated the risk of respiratory distress at birth to be four times higher for early-term newborns compared to full-term neonates [69]. McLaren et al. [70] compared early-term and full-term newborns delivered to women with multiple prior cesarean births and concluded that early-term newborns had twice the risk of requiring assisted ventilation >6 h. These emerging reports suggest a mediating role of cesarean delivery in the risk association between delivery before 39 weeks and respiratory compromise, warranting further investigation.

Late-preterm delivery interrupts the normal physiological development of the fetal respiratory system, leaving the newborn with a structurally and functionally immature lung and, hence, vulnerable to various pulmonary pathologies [71,72]. Since administering antenatal steroids during the late-preterm window significantly reduces neonatal respiratory complications [73], it is now recommended for women at risk of delivery between 34^0/7^ and 36^6/7^ weeks of gestation [74]. However, pulmonary immaturity might not be the only factor responsible for adverse respiratory outcomes for deliveries before 39 weeks of gestation. Bates et al. [75] observed that even after documentation of fetal lung maturity, newborns delivered between 36 and 38 weeks were around seven times more likely to develop respiratory distress syndrome compared to those between 39 and 40 weeks. They postulated that, compared to full-term neonates, the relative overall immaturity of newborns between 36 and 38 weeks—irrespective of lung maturity status—was likely responsible for this difference. More studies will be needed to disentangle the multifaceted pathogenesis of respiratory morbidity in these newborns.

### 5.3. Metabolic Morbidity

Late-preterm newborns more frequently develop hyperbilirubinemia compared to neonates >37 weeks (4.1 vs. 1.0%) [76]. A study conducted at the Massachusetts General Hospital reported that the risk of clinical jaundice was 95% higher in late-preterm newborns compared to term deliveries, and a quarter of late-preterm newborns required phototherapy [77]. A meta-analysis demonstrated that late-preterm newborns had an increased risk of requiring phototherapy (RR = 5.0, 95% CI: 1.7–14.6) compared to newborns delivered after 37 weeks [66]. Newborns delivered late preterm tend to have a protracted course of hyperbilirubinemia and an increased likelihood of developing kernicterus [78].

Many earlier studies compared late-preterm newborns with those delivered after 37 weeks, providing no specific data for early-term births. In 2021, Mitha et al. [55] compared the complications of late-preterm and early-term newborns with full-term controls and demonstrated an increased risk of neonatal jaundice in both groups (late-preterm, aRR 12.85, 95% CI 12.51–13.20; early-term, aRR 2.77, 95% CI 2.70–2.83). A study conducted in Brazil showed that the risk of requiring phototherapy during the first three days after birth was higher in provider-initiated deliveries at 37 (OR 3.44, 95% CI 2.29–5.17) and 38 (OR 2.01, 95% CI 1.41–2.86) weeks compared to full-term newborns [32].

Hypoglycemia and hypothermia are two other metabolic problems frequently encountered by late-preterm newborns. Compared to infants between 37 and 42 weeks of gestation, late-preterm newborns have a greater risk of hypoglycemia (OR 1.60, 95% CI 1.26–2.03) and temperature instability (OR 1.80, 95% CI 1.26–2.56) during birth hospitalization [79]. A systematic review by Teune et al. [66] reported a much higher risk of hypoglycemia (RR 7.4, 95% CI 3.0–18.1) and hypothermia (10.8, 95% CI 4.6–25.0) in late-preterm newborns compared to term infants. Deficiency of glycogen stores, subcutaneous fat, and brown adipose tissue are some of the physiological limitations of late-preterm newborns, potentially involved in the pathogenesis of these metabolic morbidities [7].

Early-term newborns, on the other hand, are also susceptible to serum glucose dysregulation, with around an eighty percent higher risk of developing hypoglycemia compared to full-term deliveries [55]. Leal et al. [32] demonstrated that both spontaneous and provider-initiated early-term deliveries are associated with an increased risk of hypoglycemia during the first two days after birth compared to full-term deliveries, indicating the relative immaturity of the glucose homeostatic apparatus even at 37 and 38 weeks of gestation [32].

The spectrum of metabolic morbidity for infants delivered before full term also encompasses feeding difficulties. Late-preterm newborns have a six-fold increased risk of exhibiting feeding problems compared to infants born at term and are more likely to experience a delay in hospital discharge due to poor feeding [66,77]. Analysis of the All Our Babies pregnancy cohort in Canada compared breastfeeding characteristics between late-preterm newborns and those delivered at ≥38 weeks of gestation [80]. Compared to their term counterparts, late-preterm infants had significantly lower rates of breastfeeding within the first 24 hours of life (78.7 vs. 97.5%, *p* < 0.001), successful first-attempt breastfeeding (50.7 vs. 75.0%, p <0.001), breastfeeding before birth hospitalization discharge (78.1 vs. 90.8%, *p* <0.001), and breastfeeding at 4 months (69.3 vs. 81.7%, *p* = 0.008). Another study by Nagulesapillai et al. [81] using the same Canadian cohort evaluated breastfeeding difficulties and exclusivity at 4 months after birth for late-preterm and term (≥37 weeks) infants. After adjusting for potential confounders, the authors highlighted that late-preterm birth was associated with increased odds of breastfeeding difficulties attributable to the baby (OR 1.72, 95% CI 1.24–2.38) and lower odds of exclusive breastfeeding (OR 0.67, 95% CI 0.46–0.97) compared to term births. The frequency of exclusive breastfeeding was also studied in Iceland, with the results indicating significantly lower rates for late-preterm newborns compared to term births during the first week after discharge from birth hospitalization and at one-month postpartum [82]. Additionally, it has been reported that late-preterm newborns have, on average, a two-month shorter breastfeeding duration compared to their term counterparts during the first year [83].

Unfortunately, all studies on breastfeeding outcomes of late-preterm infants have categorized deliveries occurring beyond 37 weeks as the reference group, and relatively fewer investigations have specifically studied early-term births. However, emerging reports have elucidated that even within the term window, delivery between 37 and 38 weeks negatively impacts breastfeeding initiation, duration, and exclusivity [32,84]. It has been shown that early-term newborns have lower breastfeeding rates at discharge from birth hospitalization than full-term neonates (67.9 vs. 70.4%) [85]. In a prospective cohort study, Noble et al. [86] concluded that apart from the breastfeeding difficulties encountered during birth hospitalization, early-term infants continue to display lower rates of high breastfeeding intensity (OR 0.33, 95% CI 0.15–0.72) and exclusive breastfeeding (OR 0.40, 95% CI 0.22–0.71) compared to full-term newborns even one month after birth. These results suggest that, analogous to the trend observed for late-preterm births, breastfeeding challenges in the early-term population continue to persist well beyond discharge from birth hospitalization, warranting further research with longer follow-ups.

### 5.4. Infectious Morbidity

Late-preterm birth predisposes newborns to a range of infectious morbidities, including bacterial sepsis, meningitis, necrotizing enterocolitis, and other infections [55,66]. In contrast, early-term neonates have not been identified to be at risk for these conditions. Teune et al. [66] reported in their meta-analysis that around twenty percent of late-preterm newborns undergo sepsis evaluation and have a five-fold increased risk of having a sepsis diagnosis compared to term infants. Although they reported low rates of meningitis (0.12%) and necrotizing enterocolitis (0.11%) in the late-preterm population, when compared to term infants, the odds were significantly higher.

Recent data from a Swedish national cohort provided further evidence for an increased risk of necrotizing enterocolitis (aRR 4.00, 95% CI 1.68–9.56), bacterial sepsis (aRR 1.50, 95% CI 1.33–1.68), and other infections (aRR 2.61, 95% CI 2.35–2.89) in late-preterm newborns compared to full-term controls [55]. Surprisingly, in this cohort, early-term birth was protective against bacterial sepsis (aRR 0.85, 95% CI 0.80–0.91) compared to full-term neonates. However, this should be interpreted with caution, considering the very low overall rates of sepsis, and needs to be explored further by future investigations.

### 5.5. Neurologic Morbidity

Neurologic complications are primarily a cause of concern for late-preterm newborns. Compared to full-term controls, late-preterm newborns are more likely to experience seizures (aRR 1.63, 95% CI 1.32–2.01), intracranial non-traumatic hemorrhage (aRR 2.58, 95% CI 1.77–3.76), and hypoxic–ischemic encephalopathy and related conditions (aRR 1.48, 95% CI 1.19–1.84) [55]. Intraventricular hemorrhage grade I–II occurs more frequently in newborns delivered late preterm than in those delivered after 37 weeks (0.17 vs. 0.02%); however, grade III–IV is a rare occurrence, even in the late-preterm population (0.01%) [66].

An epidemiologic study in France investigated the risk of poor prognosis, defined as death and/or a severe neurologic condition (ischemic encephalopathy; intraventricular hemorrhage grade III–IV and/or cystic periventricular leukomalacia and/or seizures), in infants delivered at 34–41 weeks of gestation [58]. Compared to full-term births, the adjusted relative risk gradually increased from 37 weeks (1.6, 95% CI 1.1–2.3) to 34 weeks (6.8, 95% CI 4.1–11.1) of gestation. Unlike other investigations, this study identifies delivery at 37 weeks as a potential risk factor for neurologic morbidity. However, the authors performed the analysis using a composite outcome of death and/or neurologic morbidity. Hence, future studies should consider assessing neurologic outcomes separately to discern this relationship better.

### 5.6. Hospital Readmission

Unfortunately, even apparently healthy late-preterm and early-term newborns discharged soon after birth cannot be assumed to have escaped the entrapment of neonatal morbidity. The readmission rates during the neonatal period are significantly higher for both late-preterm (3.46%) and early-term (2.06%) newborns compared to full-term neonates (1.48%) [87], and around eighty percent of these readmissions occur within five days after the initial hospital discharge [88].

A retrospective study conducted in California analyzed the incidence, risk factors, and causes of one-month hospital readmission among healthy neonates born between 34 and 42 weeks of gestation [89]. Compared to infants delivered at 39 to 42 weeks, late-preterm (OR 2.34, 95% CI 2.28–2.40) and early-term (OR 1.41, 95% CI 1.39–1.43) newborns had increased odds of being readmitted. More than half of these readmissions were due to jaundice, while other causes, including infection, temperature instability, and pulmonary and gastrointestinal complications, were responsible for the remainder. For late-preterm newborns, the study investigators identified several independent risk factors for 30-day readmission, including maternal age ≥34 years, primiparity, diabetes, chorioamnionitis, assisted vaginal delivery, and male newborn gender.

Other studies have linked the duration of the initial hospital stay with the subsequent risk of readmission for late-preterm newborns, albeit with conflicting conclusions: one identifying a short birth hospitalization stay [87] and the other describing an initial stay >3 days [90] as a potential risk factor for readmission. Future studies delineating this relationship will serve as a framework to inform guidelines regarding appropriate discharge timing for these vulnerable newborns to mitigate the risk of readmission.

### 5.7. Factors Associated with Neonatal Morbidity

Does delivery before 39 weeks in and of itself account for the complications faced by late-preterm and early-term newborns, or do the various maternal, fetal, or obstetric factors leading to earlier delivery in the first place contribute to the increased morbidity risk? There has been much speculation, and the literature has been elusive; however, the likely answer appears to be both.

Shapiro-Mendoza et al. [57] concluded that both late-preterm delivery and maternal medical conditions are independent risk factors for neonatal complications, and when combined, the risk is significantly increased. Antepartum hemorrhage, maternal diabetes, and newborn malformations have been linked to increased neurologic and respiratory morbidity risk in late-preterm newborns [91]. Moreover, late-preterm birth due to hypertensive disorders, suspected fetal growth restriction, and vaginal bleeding related to placentation disorders exacerbates the risk of neonatal morbidity [92].

More recently, an investigation of Australian singleton late-preterm and early-term newborns analyzed the factors associated with increased risk of early severe neonatal morbidity [93]. The authors identified preexisting maternal diabetes, instrumental birth, emergency cesarean birth, and newborn male sex as contributing factors of morbidity in late-preterm newborns. On the other hand, preexisting and gestational diabetes, antepartum hemorrhage, instrumental birth, elective and emergency cesarean birth, nulliparity, raised maternal BMI, and male newborn sex were factors associated with early severe neonatal morbidity in early-term newborns.

Emerging evidence also highlights the negative impact of twin delivery on the neonatal outcomes of late-preterm and early-term newborns [94]. As the list of potential exacerbating factors of adverse neonatal outcomes continues to grow, future studies investigating the interactions among these factors and also with prematurity in general are warranted. This will allow for future risk stratification of late-preterm and early-term neonates.

## 6. Neonatal and Infant Mortality

Appallingly, late-preterm and early-term newborns are at an increased risk of neonatal and infant mortality compared to their full-term counterparts, as depicted in Table 2 [95]. Teune et al. [66] demonstrated in their systematic review of 22 studies that the risk of neonatal and infant mortality for late-preterm newborns was around six-fold and four-fold higher, respectively, compared to term deliveries. A national cohort study conducted in Sweden compared the mortality rates of early-term newborns with their full-term counterparts and reported an adjusted hazard ratio of 2.18 (1.89–2.51) and 1.66 (1.44–1.92) for neonatal and post-neonatal (between 28 and 364 days of life) mortality, respectively [96]. The neonatal mortality rates continue to decrease gradually from 34 weeks (7.1 per 1000) up to 39 weeks (0.8 per 1000), mirroring the trend observed for neonatal morbidity [97]. This pattern further solidifies the construct of categorizing any delivery occurring before the 39-week hallmark as high-risk.

## 7. Long-Term Consequences

**Figure 2:**
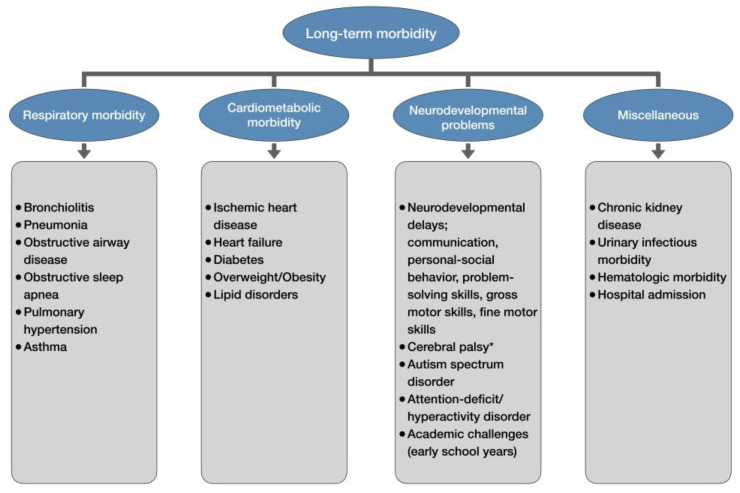
Long-term morbidity of late-preterm and early-term newborns. * only late-preterm newborns.

After mounting evidence identified late-preterm and early-term delivery as a significant risk factor for various neonatal complications, concerns for the potential long-term adverse effects of delivery before 39 weeks began to surface. Unfortunately, those concerns were well-founded, as the latest research has demonstrated associations between delivery before 39 weeks and many long-term respiratory, cardiovascular, renal, hematologic, neurologic, psychiatric, and behavioral complications in later life (Figure 2) [98].

### 7.1. Medical Morbidity and Mortality

Starting from early childhood, individuals born late preterm [99] and early term [100] have increased hospitalization rates due to bronchiolitis and pneumonia. A meta-analysis by Cahen-Peretz et al. [101] provided further evidence of increased risk of long-term respiratory morbidity, up to 18 years of age, in infants born early term compared to full-term deliveries, primarily due to respiratory infections and obstructive airway diseases. Obstructive sleep apnea also occurs more frequently in individuals (up to 18 years of age) born late preterm and early term compared to full-term births [102].

A Swedish population-based cohort study followed around three million individuals from the age of 1 year up to a maximum age of 30 to record the incidence of pulmonary hypertension [103]. Compared to individuals born at 39 weeks, those delivered at 32–36 weeks (aHR 3.42, 95% CI 2.46–4.74) and 37–38 weeks (1.74, 1.31–2.32) were at a greater risk of having a diagnosis or death from pulmonary hypertension. Another national cohort study conducted in Sweden reported an elevated risk of asthma from childhood into mid-adulthood for late-preterm (31% increased risk) and early-term (13% increased risk) births compared to deliveries between 39 and 41 weeks [104].

Children as young as 3 to 12 years of age, born late preterm, have been shown to display higher scores of cardiometabolic risk compared to those delivered full term, raising fears of potential adverse cardiovascular events in adulthood [105]. These concerns were recently validated when the analyses of the Swedish national birth cohort, following individuals up to a maximum age of 43 years, yielded statistically significant hazard ratios for individuals born both late preterm and early term for the outcomes of ischemic heart disease [106] and heart failure [107], compared to individuals born at 39–41 weeks.

Studies have also identified late-preterm [108] and early-term [109] delivery as risk factors for diabetes in early adulthood. Additionally, children born early term are more prone to obesity compared to full-term births [109]. Both late-preterm and early-term births have also been identified to modestly increase the risk of lipid disorders in early adulthood compared to deliveries at 39–41 weeks [110].

Delivery before full term also appears to influence the renal, urinary, and hematologic systems. Padeh et al. [111] recorded the rates of hospitalization of infants up to 18 years of age due to infectious urinary morbidity. Their results indicated that both late-preterm and early-term deliveries were independently associated with urinary tract infections when compared with full-term births. Like other chronic diseases, the risk for the development of chronic kidney disease is also increased by late-preterm (84% increased risk) and early-term (30% increased risk) delivery compared to full-term birth, possibly indicating the interruption of normal fetal kidney development [112]. Another cohort analysis focusing on hospitalizations up to 18 years of age due to hematologic morbidity demonstrated that early-term delivery modestly increased the risk of such hospitalizations compared to full-term birth [113].

Mirroring the trend of neonatal and infant mortality, delivery before full term continues to serve as a risk factor for mortality well into adulthood. The Swedish national birth cohort study revealed that the adjusted hazard ratio for all-cause mortality from birth to age 45 years was 2.61 (95% CI: 2.52–2.71) for late-preterm delivery and 1.34 (1.30–1.37) for early-term delivery compared to full-term birth [17]. These ratios reduced to 1.22 (1.07–1.39) for late-preterm delivery and 1.16 (1.08–1.25) for early-term deliveries for mortality at age 30–45 years; however, they remained significantly higher than full-term birth.

Similar results were also furnished by a multinational cohort study conducted in four Nordic nations, where the risk of all-cause mortality after the age of 15 years was 23% higher in individuals born late preterm and 12% higher in those born early term compared to full-term births [18]. The authors also conducted a cause-specific mortality analysis, which demonstrated an elevated risk for noncommunicable disease and cardiovascular disease mortality for both these infant populations when compared with individuals delivered full term.

These alarming revelations underscore the significance of the long-term follow-up of individuals delivered before full term. Adult healthcare providers should be specifically trained to view these individuals with a high index of suspicion for various adult chronic diseases and mortality.

### 7.2. Neurodevelopmental Outcomes

The relative immaturity of brain development in infants delivered before 39 weeks becomes evident as early as within the first 6 months of life, with late-preterm and early-term infants displaying a significantly higher risk of neurodevelopmental delay compared to deliveries occurring after 39 weeks [114]. It has been reported that from 41 weeks to 34 weeks of gestation, each week of decreasing gestational age increases the risk of developmental delay by 84% in children aged between 1 and 5 years [115]. Children born late preterm and early term are more likely to be classified as having suspected developmental coordination disorder compared to those born full term [116]. A critical review including 35 studies illuminated that in addition to motor delay and coordination disorder, late-preterm delivery also elevated the risk of cerebral palsy when compared to full-term births [117]. Table 3 illustrates the range of neurodevelopmental complications associated with late-preterm and early-term birth [118].

A Chinese national cohort study evaluated the neurodevelopment of 137,530 children aged 3–5 years using the Ages and Stages Questionnaire-Third Edition (ASQ-3) [119]. After adjusting for potential confounders, children born late preterm and early term were more prone to neurodevelopmental delays across all five domains (communication, personal-social behavior, problem-solving, gross motor skills, and fine motor skills) compared to their full-term counterparts. Analysis of the Japan Environment and Children’s Study database, studying children aged 3 years using the ASQ-3 questionnaire, yielded similar results, with the exception that early-term birth was not associated with personal-social behavior and problem-solving domains of neurodevelopmental delay in this cohort [120]. However, it is worth highlighting that Japanese early-term infants at 6 and 12 months of age have been shown to perform poorly across all domains of ASQ-3—including personal-social behavior and problem-solving—compared to 40-week deliveries, as reported by Haneda et al. [121] using the same Japan Environment and Children’s Study database. This likely suggests that the neurodevelopmental outlook for children born early term could potentially improve with age.

Other investigators have focused more on the combined neurodevelopmental outcomes of moderate-to-late-preterm (32–36 weeks) delivery. An Australian prospective cohort study concluded that children aged 9 years born moderate-to-late preterm had lower full-scale IQ scores, displayed poorer performance in verbal comprehension, visuospatial, and working memory domains of cognition, and were more prone to behavioral difficulties compared to term births [122]. Additionally, this study highlighted an association between developmental delays at the age of 2 years and adverse neurodevelopmental outcomes at the age of 9 for moderate-to-late-preterm children. Palomero-Sierra et al. [123] compared language development between moderate-to-late-preterm and full-term births. At 12 months of age, moderate-to-late-preterm children scored poorly only in cognition, whereas differences in receptive and expressive language development between the two infant groups appeared at 18 and 24 months, respectively. Lately, children born moderate-to-late preterm have also been identified to lag behind term births in executive function. A meta-analysis of 12 studies reported poorer scores for deliveries at 32–36 weeks compared to term birth not only in overall executive function but also in all its individual domains, namely attentional control, cognitive flexibility, and goal setting [124].

For all the aforementioned outcomes, future investigators should also consider separately analyzing the late-preterm and early-term populations while using full-term controls. Recently, Nivins et al. [125] used the Adolescent Brain and Cognitive Development Study cohort to compare a wide range of cognitive outcomes in children born at various gestational age subgroups, including late preterm and early term, with deliveries ≥39 weeks. Unlike previous investigations, the authors decided to adjust for genetic factors believed to be related to neurodevelopment in addition to other potential confounders. Surprisingly, in this adjusted analysis, children born late preterm and early term did not differ from their full-term counterparts, while the association between moderate preterm (32–33 weeks) birth and poor cognitive outcomes remained statistically significant. These results offer insights into how genetic factors may be linked to neurodevelopment and confound relationships between earlier delivery and neurodevelopmental challenges. Perhaps it would be wise to account for genetics in future investigations to arrive at more definitive conclusions regarding the independent effect of late-preterm and early-term delivery on neurodevelopmental delays.

Emerging evidence has also shown that the incidence of neuropsychiatric disorders is higher in individuals born late preterm (17%) and early term (7%) compared to full-term births [126]. Analyses of the Swedish national birth cohort data of 1973–2013 revealed an increased prevalence of autism spectrum disorder (ASD) and attention-deficit/hyperactivity disorder (ADHD) in individuals born late preterm (ASD: 1.9%; ADHD: 5.7%) and early term (1.6% and 5.2%) compared to those delivered full term (1.4% and 4.5%) [127,128]. Data from the Taiwan Maternal and Child Health Database further elucidated that the risk of ASD with co-occurring intellectual disability was around 40% higher in children born late preterm compared to those delivered between 37 and 41 weeks [129]. Another investigation of teacher-reported ADHD symptomatology in children aged 9 years concluded that compared to students born at full term, early-term peers had 23% and 17% higher scores for hyperactivity and ADHD, respectively [130].

Given the diversity of neurodevelopmental complications of late-preterm and early-term delivery, follow-up care for these disorders starting from early childhood should be incorporated into routine practice. This will allow for early detection and timely intervention to manage these conditions.

### 7.3. Educational Outcomes

As opposed to the belief that neurodevelopmental effects of late-preterm and early-term delivery are transient and that these children potentially catch up with their full-term counterparts by the time of school entry, emerging evidence somewhat discredits this assumption. A systematic review by Chan et al. [131] highlighted that late-preterm and early-term birth was associated with poorer performance in general cognitive and school language tests, increased special educational needs, and increased likelihood of failure to complete secondary and postsecondary education. Further evidence of increased utilization of special education during primary school years by children born at 32–36 weeks (aOR: 1.61; 95% CI: 1.57–1.65) and those delivered at 37–39 weeks (1.18; 1.17–1.20) compared to 40-week controls was provided by a recent population-based matched cohort analysis conducted in the Netherlands [132].

Other studies have demonstrated that compared to full-term births, students born late preterm are more likely to repeat kindergarten or grade 1 [133], whereas those delivered early term display poorer language and literacy skills [134] and score lower in reading and mathematics [135].

However, the Bradford birth cohort yielded results illuminating that children aged 5–11 years, born early term, did achieve the expected levels of overall educational achievement comparable to those born full term [136]. It further revealed that although late-preterm deliveries were at an increased risk of failing to achieve this educational milestone at the ages of 5 and 7 years, the differences became insignificant at 11 years. The possibility of catch-up in school performance during the later years of school was further strengthened by Alterman et al. [137], who demonstrated that despite the differences observed in primary school, the likelihood of performing well in secondary school was comparable for deliveries ≥32 weeks of gestation and full-term births.

Nevertheless, awareness among educational providers of the potential academic challenges faced by late-preterm and early-term children during the early school years is crucial for mitigating these educational problems.

## 8. Discussion and Future Research Directions

Literature has made it abundantly clear that late-preterm and early-term deliveries cannot be viewed as risk-free. It is now evident that, apart from predisposing newborns to neonatal morbidity and mortality, delivery before 39 weeks continues to serve as a risk factor for complications extending well into adulthood. Hence, any conflation of these births with full-term deliveries should be strongly discouraged.

Given the rapid advancement of knowledge concerning late-preterm and early-term births, this review has attempted to incorporate all recent insights while reiterating previously known facts regarding these deliveries. Studies conducted in the past few years have significantly expanded our understanding regarding the spectrum of complications faced by these newborns, unveiling many new adverse outcomes previously unknown to be linked to late-preterm and early-term delivery. These mainly include long-term medical morbidities like pulmonary hypertension [103], cardiovascular disease [105,106,107], lipid disorders [110], chronic kidney disease [112], and even mid-adulthood mortality [17,18]. While concerns regarding late-preterm infants exhibiting developmental delays have long existed, it was not until recently that emerging evidence has unraveled the sheer magnitude and diversity of neurodevelopmental impairments encountered not only by late-preterm but also early-term newborns [114,115,118]. The risk of neuropsychiatric disorders like autism and ADHD, once thought only to be associated with preterm birth, has now also been linked to early-term deliveries [127,128]. Newer investigations focusing on neonatal morbidity of late-preterm and early-term newborns have not only strengthened previous evidence of the heightened risk of various neonatal complications but also unearthed a multitude of factors exacerbating this risk [62,68,69,70,91,92,93,94], providing invaluable information for risk stratification and preventive strategies. Similarly, while the increased incidence of hospital readmissions for these newborns became evident several years ago [87,88], the causes and risk factors for these readmissions have only recently begun to appear in the literature [89,90]. Lastly, various authors have recently elucidated specific risk factors leading to late-preterm and early-term birth [43,44,45,46,48,49,50,51,52,53,54]—a departure from the prior custom of studying the causes of preterm birth in general.

All this new information regarding late-preterm and early-term births will be essential for healthcare professionals to anticipate risks, devise mitigation strategies, and tailor effective treatment options for these vulnerable newborns. However, despite the newer insights offered, the current evidence base is not without its limitations, and several research questions remain unexplored.

Although the neonatal course of late-preterm newborns has been well documented, relatively fewer studies have specifically analyzed the neonatal outlook of early-term deliveries in the full breadth of neonatal morbidity. Perhaps the relatively recent introduction of the descriptor ‘early term’ in neonatal research is responsible for this disparity and explains the frequent designation of control groups as ≥37 weeks in earlier studies. Moreover, the last decade saw a paucity of research on the birth hospitalization outcomes of late-preterm and early-term newborns, with authors’ interest shifting more toward long-term complications. Considering the changing obstetric practices and rising birth rates of these newborns over the past decade [23,24,27,30], the potential impact of these changes on the neonatal complications of these infants needs to be explored, and newer studies—with full-term controls—are warranted to reflect the current scenario.

Efforts should also be directed to stimulate high-quality research in low- and middle-income nations. Perhaps our most significant limitation in estimating the true global impact of late-preterm and early-term newborns is the dearth of data offered by the developing world. Unless data from these parts of the world is incorporated into the medical literature, all our risk estimates for these infants will potentially remain an underestimation of the actual risk.

Prospective authors should also consider analyzing the outcomes of newborns by each individual gestational week, in addition to the broad categorization of late-preterm and early-term groups. This is because evidence has demonstrated that classifying all late-preterm and early-term newborns within the same risk category is somewhat misleading, given that complication rates vary by each gestational week [57,58]. This is particularly relevant for early-term newborns since some studies have shown that deliveries occurring at 38 weeks are not at risk for specific neonatal morbidities, unlike those occurring at 37 weeks [58,60,64].

Future studies should also attempt to disentangle the complex interaction of various maternal, fetal, and obstetric factors mediating the risk association of prematurity and neonatal morbidity to allow for risk stratification of newborns within each gestational age subgroup. Unveiling the full spectrum of risk factors can also help formulate preventive strategies to counter neonatal complications and improve the quality of healthcare offered to these vulnerable newborns.

Long-term complications of late-preterm and early-term delivery have been mostly studied in Scandinavia, particularly Sweden, leaving us with prevalence estimates that are more reflective of the Nordic population than the global populace. Research in this domain should be facilitated in other parts of the world to unravel the global extent of the long-term morbidity associated with these deliveries and elucidate the spectrum of mediating factors acting differently across populations. Moreover, the current analyses are limited in their follow-up duration of up to early adulthood (<50 years) [17,106,107], with the outcomes of late-preterm and early-term delivery in late adulthood being virtually unknown. Future investigations with longer follow-ups will be crucial in providing insights into the effects of delivery before full term during the later years of life.

With that said, the current literature provides compelling evidence for the need to think differently about late-preterm and early-term infants. Obstetric bodies bear the onus of formulating new delivery guidelines in light of the emerging short- and long-term complications of delivery <39 weeks. On the other hand, pediatric practitioners and policymakers should exercise caution that the plight of preterm newborns delivered at <34 weeks does not hinder adequate healthcare resource allocation for deliveries occurring after 34 weeks, especially in resource-limited settings. Although the rates of complications increase even further as gestational age drops below 34 weeks [4,5,17], the newborn birth rates after 34 weeks are comparatively much higher [1,23,30], exerting a public health burden of far greater proportions.

## 9. Conclusions

Late-preterm and early-term newborns are the fastest-growing subgroups in neonatology, representing a grave public health challenge. They share many of the same risk factors as preterm birth in general. Compared to full-term delivery, late-preterm and, to a lesser extent, early-term birth increase the risk of various neonatal complications, including NICU admission, respiratory distress syndrome, transient tachypnea of the newborn, pneumonia, invasive and non-invasive ventilation, jaundice, hypoglycemia, breastfeeding difficulties, and increased rates of hospital readmission. On the other hand, neurologic and infectious morbidities are predominantly associated with late-preterm delivery. Many maternal, fetal, and obstetric factors have been identified as playing a mediating role in the neonatal complications of late-preterm and early-term newborns. Apart from complications encountered during birth hospitalization, late-preterm and early-term infants more frequently acquire childhood respiratory and urinary infections, with higher hospitalization rates than their full-term counterparts. Long-term sequelae of late-preterm and early-term birth encompass many chronic illnesses like diabetes, cardiovascular disease, chronic kidney disease, asthma, and lipid disorders. Moreover, these newborns are more likely to display neurodevelopmental delays, behavioral and neuropsychiatric problems, and academic challenges. In addition to the neonatal and infant mortality risk, late-preterm and early-term birth elevate the risk of mortality well into mid-adulthood, underscoring the importance of recognizing these newborns as high-risk.

## Figures and Tables

**Table 1:** Risk factors for late-preterm and early-term birth.

Maternal factors • Diabetes mellitus • Hypertension • Eclampsia/preeclampsia • Advanced age • Low BMI • Drug abuse • Trait anxiety
Obstetric factors • Placentation disorders • Poly-/oligohydramnios • Previous preterm/early-term delivery • Assisted pregnancy • Interpregnancy interval <12 months • Cesarean delivery
Environmental factors • Antenatal exposure to air pollutants • Antenatal exposure to high ambient temperature

**Table 2:** Risk of neonatal and infant mortality of late-preterm and early-term newborns.

	Neonatal Mortality	Infant Mortality
Gestational Age (Weeks)	RR (95% CI)	RR (95% CI)
34	12.0 (6.6–21.7)	10.3 (6.8–15.5)
35	10.1 (6.3–16.2)	6.5 (4.4–9.5)
36	6.2 (4.1–9.5)	4.2 (3.0–5.9)
37	2.5 (1.6–3.9)	2.6 (1.9–3.5)
38	1.3 (0.8–1.9)	1.4 (1.0–1.8)
39	0.9 (0.6–1.3)	1.0 (0.8–1.4)
40	reference	reference

(Data from [95]).

**Table 3:** Risk of various neurodevelopmental impairments in children born late preterm and early term compared to full-term birth.

Neurodevelopmental Impairment	Late-Preterm	Early-Term
	HR (95% CI)	HR (95% CI)
Motor	1.90 (1.70–2.13)	1.28 (1.20–1.38)
Cognitive	1.31 (1.24–1.39)	1.14 (1.10–1.17)
Epileptic	1.23 (1.12–1.36)	1.06 (1.01–1.11)
Visual	1.42 (1.32–1.52)	1.10 (1.05–1.14)
Hearing	1.16 (1.08–1.25)	1.04 (1.00–1.08)
Composite ^1^	1.30 (1.26–1.35)	1.08 (1.06–1.11)
Severe/Major ^2^	1.55 (1.40–1.72)	1.10 (1.03–1.17)

^1^ One or more of the following impairments: motor, cognitive, epileptic, visual, or hearing. ^2^ Cerebral palsy, severe intellectual disability, generalized epileptic disorder, or severe hearing/visual impairment. (Data from [118]).

## Data Availability

The original contributions presented in this study are included in the article.

## Abbreviations

The following abbreviations are used in this manuscript:

(a)RR	(adjusted) relative risk
CI	confidence interval
(a)OR	(adjusted) odds ratio
(a)HR	(adjusted) hazard ratio
